# The Environmental and Economic Sustainability of Carbon Capture and Storage

**DOI:** 10.3390/ijerph8051460

**Published:** 2011-05-09

**Authors:** Paul E. Hardisty, Mayuran Sivapalan, Peter Brooks

**Affiliations:** 1 WorleyParsons EcoNomics™, Perth, WA 6000, Australia; 2 Imperial College London, London SW7 2AZ, UK; 3 WorleyParsons EcoNomics™, Houston, TX 77079, USA; E-Mail: mayuran.sivapalan@worleyparsons.com; 4 WorleyParsons, Brisbane, QLD 7000, Australia; E-Mail: peter.brooks@worleyparsons.com

**Keywords:** CO_2_, greenhouse gas, CCS, geosequestration, LNG, climate, sustainability, economics

## Abstract

For carbon capture and storage (CCS) to be a truly effective option in our efforts to mitigate climate change, it must be sustainable. That means that CCS must deliver consistent environmental and social benefits which exceed its costs of capital, energy and operation; it must be protective of the environment and human health over the long term; and it must be suitable for deployment on a significant scale. CCS is one of the more expensive and technically challenging carbon emissions abatement options available, and CCS must first and foremost be considered in the context of the other things that can be done to reduce emissions, as a part of an overall optimally efficient, sustainable and economic mitigation plan. This elevates the analysis beyond a simple comparison of the cost per tonne of CO_2_ abated—there are inherent tradeoffs with a range of other factors (such as water, NOx, SOx, biodiversity, energy, and human health and safety, among others) which must also be considered if we are to achieve truly sustainable mitigation. The full life-cycle cost of CCS must be considered in the context of the overall social, environmental and economic benefits which it creates, and the costs associated with environmental and social risks it presents. Such analysis reveals that all CCS is not created equal. There is a wide range of technological options available which can be used in a variety of industries and applications—indeed CCS is not applicable to every industry. Stationary fossil-fuel powered energy and large scale petroleum industry operations are two examples of industries which could benefit from CCS. Capturing and geo-sequestering CO_2_ entrained in natural gas can be economic and sustainable at relatively low carbon prices, and in many jurisdictions makes financial sense for operators to deploy now, if suitable secure disposal reservoirs are available close by. Retrofitting existing coal-fired power plants, however, is more expensive and technically challenging, and the economic sustainability of post-combustion capture retrofit needs to be compared on a portfolio basis to the relative overall net benefit of CCS on new-build plants, where energy efficiency can be optimised as a first step, and locations can be selected with sequestration sites in mind. Examples from the natural gas processing, liquefied natural gas (LNG), and coal-fired power generation sectors, illustrate that there is currently a wide range of financial costs for CCS, depending on how and where it is applied, but equally, environmental and social benefits of emissions reduction can be considerable. Some CCS applications are far more economic and sustainable than others. CCS must be considered in the context of the other things that a business can do to eliminate emissions, such as far-reaching efforts to improve energy efficiency.

## 1. Introduction

### 1.1. Background—Technology, Policy, Economics

Carbon capture and storage (CCS) is one of a host of technical solutions that are currently available for reducing global emissions of greenhouse gases (GHG) to the atmosphere, and thus curb the longer term effects of anthropogenic climate change. However, the barriers to solving the world’s climate change challenge are not, in the main, technical. There is now widespread scientific consensus on the major causes of climate change [1], the overall planetary risks of inaction, and even on the combinations of measures which will be required to deliver the emissions reductions required to eliminate the worst of the future risks posed by an earth system in flux [2]. Reductions in emissions will be required in a wide range of sectors, including from land-use changes (forest clearing and agricultural practices), building design and operation, transport, and notably electrical power generation [3]. Research and technical development has been underway in all of these areas for many years now, and a wide range of technically viable and workable solutions already exists in each of these sectors.

Indeed, the broad scientific and policy-making community has been aware of the risks of climate change, and the solutions available, for well over two decades [4]. However, during that same period, on a global scale, the tangible effects of actions taken to reduce emissions of greenhouse gases (GHG) have been minimal. Not only has the world dramatically increased its consumption of all kinds of energy over the last thirty-five years, our energy mix has also remained essentially unchanged [5]. The proportion of overall energy requirements provided by fossil fuels has actually increased over this period, despite full and growing knowledge of the risks posed by climate change. On a global scale, we have, in effect, done little so far to combat it. Why?

Climate change is a global issue, and does not respect national boundaries. Nor does it care about public opinion. We understand the science of the problem, at least in overall terms, if not in detail. We have the technology now to significantly reduce emissions. What has been lacking, so far, is the political, social, and economic will to deploy these existing technologies at scale. Action has been slow and insufficient because we do not want to pay the price of action. So we wait for the cost of abatement to drop, hoping that a new low-cost technology will be developed which can do the job. But there is a fundamental difference between the value of something and its price. Dealing with climate change may come at a relatively high price. But what matters are the benefits that result from that expenditure, not only to the emitters, but to society as a whole.

### 1.2. The Economics of Climate Change

Recently, there have been several attempts to quantify a global perspective on the economics of action and inaction on climate change. These studies have considered the costs of action to reduce emissions, and the economic costs that would result if climate change is allowed to take its course unhindered. A summary of the results of recent studies is provided in Table 1.

These studies largely focus on the benefits (as damage avoided) to the man-made economy of climate change mitigation. Associated benefits from the elimination of other types of air-emissions that would occur as part of GHG reductions (lower emissions of particulates, soot, NOx and SOx from power plants, for instance), and the value of benefits of biodiversity and ecosystem protection, for instance, are rarely considered, but could be significant.

Nevertheless, it is clear, from a macro-economic perspective, that the world is far better off with concerted action to achieve deep cuts in GHG emissions than it would be otherwise. However, our global economic system is not set up to either measure or reward firms (or individual countries) for acting in the common good. Firms of all kinds must seek to maximise profit so that they can remain in business and deliver shareholder returns. Society, and the governments that represent them, must therefore regulate the activities of the market to achieve desired social outcomes. Hence, widespread discussions are currently underway worldwide to put in place mechanisms which will effectively put a price on carbon emissions to the atmosphere.

Reducing emissions from electrical power generation is one of the most important steps than can be taken in an overall GHG mitigation effort. Electricity production contributes approximately 25% of the total of direct man-made GHG emissions today [9]. The widespread adoption of coal-fired power, especially in the rapidly developing economies of China and India, is predicted to significantly increase the overall emissions from this sector over the next twenty years. Given coal’s abundance and low cost, it is unlikely that its use can be radically curtailed any time soon, no matter how quickly climate change affects the planet, or how rapidly the world responds.

On this basis, there is now significant agreement among policymakers in many countries that carbon capture and geo-sequestration (CCS) has a vital role to play in the overall efforts to reduce GHG emissions worldwide. In particular, our ability to retrofit existing coal-fired power plants, and to retro-fit other types of high-emission facilities with post-combustion capture, will be essential if we are to meet desired atmospheric stabilisation targets. The problem is that capture and geologic storage of CO_2_ is generally considered to be expensive.

## 2. CCS in Perspective

CCS has been widely identified as a significant potential contributor to global strategies aimed at reducing emissions of GHG to the atmosphere. Much of the focus on CCS to date has been in the area of government funded research and development, both in terms of capture technology, and in studying the long term fate and mobility of CO_2_ in various subsurface environments. In particular, CCS has been seen as a way to significantly reduce the GHG impacts of the widespread global use of coal for electrical power generation. On this basis, governments in several developed nations (such as the USA, Canada, Australia and some in Europe) continue to fund a range of demonstration projects designed to prove the technology, develop operational experience, and spearhead the drive to cost-efficiency. The technical feasibility of each of the individual components of CCS (capture, transport and geological sequestration) is well understood. In the gas industry, amine systems for removing entrained CO_2_ in raw gas have been widely used for years. The basic technology is mature and robust. Equally, the transport of CO_2_ via pipeline is well understood. Thousands of kilometres of CO_2_ pipeline systems have been laid and operated, much of it associated with dedicated enhanced oil recovery operations. The geo-sequestration element of CCS is the least well-developed of the three components, but nevertheless the petroleum and waste management industries have decades of experience in injecting fluids of all types into geological formations for long-term storage. Nevertheless, there continues to be significant public opposition and concern about the risks associated with long term CO_2_ leakage from storage sites [10]. It is perhaps in the combination of all of these elements into a fully-integrated project that the main challenges for CCS arise.

Globally, there are 62 active or planned commercial scale integrated CCS projects, comprising capture, transport and sequestration elements, sequestering over 1 Mtpa CO_2_ [11]. Of these, however, only seven projects are currently in the operational stage; the remainder are in the evaluation, definition, or execution stages. To date, it has been in the petroleum industry that much of this full-scale operational application of CCS has occurred: six of the projects are at natural gas processing facilities and, of those, two are offshore. As will be discussed below, much of the reason for the leadership of the gas sector in CCS is that the marginal cost of applying CCS in this sector is generally significantly lower than in other sectors, particularly coal-fired power generation [12].

## 3. CCS Economics

The overall life-cycle environmental, social and economic sustainability of CCS is examined through considering three different applications: managing the CO_2_ entrained in reservoir gas in the natural gas sector; retro-fit of CCS to stationary fixed coal-fired power generation; and reducing the GHG footprint of a liquefied natural gas plant (LNG). In these examples, the Environmental and Economic Sustainability Assessment (EESA) method is used, in which various options are considered by not only examining conventional financial costs of abatement, but also explicitly valuing the environmental and social externalities affected by each option [13]. While carbon emissions to atmosphere are clearly the major externality, other external costs and benefits also exist. In this analysis, a sustainable and economic solution is one which generates more benefit than cost, to all stakeholders, when all environmental, social and economic factors are considered across the full life cycle.

### 3.1. Managing CO_2_ Entrained in Natural Gas

The natural gas industry currently leads the commercial scale application of integrated CCS (>1 Mtpa) worldwide. Two of the largest and most successful projects have been offshore, both in Norway, where a significant and long-standing carbon pricing mechanism (since 1991) has helped to drive development of CCS.

The Snøvit project, led by Statoil, has been in production since October 2007 and currently produces approximately seven billion cubic metres of gas per year from an offshore field. CO2 is removed from the gas stream and piped about 150 km back to the field for injection through a dedicated well. Since April 2008, around 0.7 Mtpa of CO_2_ has been safely injected and stored in the Tubåen sandstone (some 2,600 metres beneath the seabed). A monitoring program has been set-up to investigate the behaviour of CO_2_ underground.

The Statoil Sleipner project, also in the North Sea off the coast of Norway produces natural gas with about 9% CO_2_, which is too high for customer requirements. By capturing some of the CO_2_ from the reservoir gas, the CO_2_ level is reduced to 2.5% to meet export and customer specifications. The Sleipner capture and storage gas processing facility, operational since 1996, is one of the global pioneers of CCS. It is the world’s first fully operational offshore gas field with CO_2_ injection. It is also the world’s first CO_2_ storage project in a geological formation 1000 metres below the sea floor. Approximately 1 Mtpa of CO_2_ is separated from produced gas and injected into a saline aquifer above the hydrocarbon reservoir zones. Maximum injection is planned for 20 Mt, with 8 Mt injected to date.

Recent reviews have shown that the cost of CCS for CO_2_ entrained in the raw reservoir gas is low compared to other applications. The Global Carbon Capture and Storage Institute found that on average, CCS increased the cost of production for stationary power production by between 39% (for IGCC) and 78% (for supercritical pulverized coal plants). However, in natural gas processing, CCS increased cost of production on average by only 1% (the lowest of all sectors). CO_2_ capture is inherent in the design of gas processing facilities where the reservoir gas contains carbon dioxide, so the marginal cost of CCS is only in the transport and sequestration components, which typically represent less than 20% of the total cost of CCS. Pipelining costs for a single facility are typically in the range of USD 3 to 4/t CO_2_ (depending of course on the distances involved), and geo-sequestration costs are in the order of USD 3 to 8/t CO_2_. Initial reservoir identification and characterization costs are typically in the range of USD 25 m to 150 m. The overall costs of CCS are likely to come down in future as CCS is more widely deployed worldwide.

For an offshore natural gas facility operating in Australia with 12% CO_2_ in reservoir gas, CCS was examined as part of a wider GHG management strategy. The availability of a well characterized down-dip part of the producing reservoir for geo-sequestration added significantly to the overall technical feasibility of CCS. The average cost of CCS at this facility was estimated to be in the order of USD 15 to 25/t CO_2_ over the project life-cycle. Other recent studies have suggested that CCS for CO_2_ in gas processing ranges from about USD 18/t CO_2_ to as much as USD 40/t CO2. Given that the Australian Carbon Pollution Reduction Scheme (abandoned in 2008) was predicted to generate an effective carbon price of as much as USD 40/t CO_2_e by 2010, and that the current government is discussing the imposition of a carbon tax in the near future, and considering that the current social cost of carbon is likely in the range of USD 50 to 100/t CO_2_e right now, CCS for this application may already be economic (from society’s perspective), and is very likely to be financially advantageous for project proponents within the near to medium term (in this discussion, economic refers to the full environmental, social and financial perspective, while financial refers only to the costs and benefits to the project proponent).

### 3.2. Coal-Fired Power Station Retrofit

An existing coal-fired power station in Australia was examined in a detailed engineering, economic and sustainability feasibility study to determine the practicality of applying CCS to dramatically reduce GHG emissions. The 25-year old 425 MW facility currently produces about 2 Mtpa of CO_2_. Two capture scenarios were investigated: A 5500 tpd system which would capture the balance of emissions at typical operating loads, and a second, larger system (8200 tpd) designed to capture 100% of flue gas when the plant’s boilers are operating at maximum continuous rate. The analysis considered all aspects of the retrofit, including plant layout and access, capture technology selection, transport of CO_2_, and identification of suitable disposal sites, within a context of what can be achieved today, with existing technology, knowledge and resources.

#### 3.2.1. Capture

A wide range of currently available capture technologies was considered. As shown in Table 2, not all are applicable to post-combustion capture, and not all are commercially available. On this basis, and because of the limited process information available for most of the other technologies, the study was based on a monoethanolamine capture technology, which is both commercially available through a number of vendors, and is fully applicable to the large-scale retrofit being considered. Installation of the CO_2_ capture system and compression at the plant will require an area of about 3,000 m^2^ for the smaller option, and 4,500 m^2^ for the larger capacity option.

#### 3.2.2. Performance

Installation of the capture system has a significant effect on the performance of the plant. The monoethanolamine system puts a significant additional energy demand on the plant. Table 3 exhibits one of the ironies of CCS in this application: to capture CO_2_, significantly more coal must be burned. Nevertheless, option 2 reduces overall annual emissions from 2 Mt to about 0.25 Mt.

#### 3.2.3. Transport and Storage

A permitted and available geo-sequestration site was assumed to exist approximately 500 km from the power station. Transport of CO_2_ overland by pipeline was assumed. Pipeline is the established method for moving large volumes of CO_2_ over long distances. Most of the current expertise in CO^2^ transport lies in the petroleum industry, where CO_2_ is widely used for enhanced oil recovery. Over 4,000 km of dedicated CO_2_ pipelines are currently in operation in the USA [14].

Two scenarios were considered: one where a dedicated pipeline transports 3 Mtpa of CO_2_ to the sequestration site, and another where several operators share a larger 12 Mtpa transport and sequestration system and share the associated economies of scale. Compression power of 40 MW is provided at the plant.

#### 3.2.4. Cost Analysis

The total capital outlay for this CCS retrofit was estimated to range between about USD 0.5 bn and USD 1 bn, paid out over a seven-year construction period. Table 4 shows the range of estimated unit costs per tonne of CO_2_ avoided for CCS retrofit, in present value terms over an assumed operations life of 25 years, using a 10% discount rate. The least preferred scenario included dedicated infrastructure, poor sequestration reservoir performance, and high estimates for capture costs, while the preferred scenario involved shared infrastructure (with unit costs approximately one-third lower than dedicated infrastructure), low-end estimates of capture costs and optimal reservoir performance. Of these aggregated unit costs, about 80% of the cost was for capture, 10% for transport and 10% for sequestration. It is also important to note that the highest uncertainty in cost was associated with the sequestration component. The range of unit cost estimates reflects the commercial and engineering uncertainty inherent in delivering a complete CCS project at the present time.

#### 3.2.5. External Costs and Benefits

This analysis, for a real facility, using technology available now, shows that under present policy positions, retrofitting existing coal-fired power stations is a not financially viable proposition for operators. The main benefit of employing CCS is to create and environmental and social benefit associated with reducing carbon emissions to the atmosphere. The value of this benefit is expressed as the social cost of carbon, or the real value of the damage caused to society by each additional tonne of GHG emitted to the atmosphere. Using high estimates of the social cost of carbon, such as Stern’s USD 85/t CO_2_e, this CCS retrofit would be marginally beneficial using option 1 of the preferred scenario. If the social cost of carbon were to rise over time (as it will if global emissions are not curbed), then preferred scenario retrofit becomes increasingly beneficial from society’s perspective.

However, until an effective price on carbon exists, operators have no financial incentive to deploy CCS. In this example, carbon price would have to reach at least USD 75/t CO_2_e, or the costs of CCS would have to drop dramatically, before this operator could justify a CCS retrofit. A much more advantageous approach for this operator in the near term would be to examine other alternatives to removing carbon emissions from its overall portfolio, where this can be achieved at lower cost. This might include examining new build plants using more efficient super-critical designs, and pre-combustion options located closer to disposal sites.

While carbon emission reduction is the chief benefit of CCS, removal of other air pollutants such as oxides of nitrogen and sulphur, particulates, and even heavy metals, may also occur as a result of capturing and treating effluents. The valuation of these additional atmospheric benefits is discussed in more detail in the following example.

In addition, there is also a range of potential external costs associated with deep geological disposal of CO_2_. Risks associated with geo-sequestration of CO_2_ include gas leakage to surface in populated areas (7% to 10% CO_2_ in air is sufficient to cause immediate danger to human life and health), acidification of groundwater supplies, and geological instability [15]. Any of these eventualities could generate significant external social and environmental costs. The likelihood and magnitude of these risks will vary considerably depending on the geological conditions of the reservoir, location, nearby population density, and the vulnerability of nearby aquifers [16]. Proper sequestration site selection, design and monitoring can significantly reduce the risk of leakage and the severity of impact should leakage occur [17]. All of these external costs would make CCS more costly from an overall environmental, social and economic perspective, over the long term. Only if the social cost of carbon is sufficiently reflected in an effective price for carbon, and if it rises significantly over time, will the operator, in this example, be able to justify deploying CCS.

### 3.3. Reducing the GHG Emissions from LNG Manufacture

LNG operations release GHG emissions at various stages of production, shipping, re-gasification, storage and distribution, including consumption. This example focuses on CCS applied to managing CO_2_ emissions from a typical LNG plant. Given the predominance of cost associated with carbon capture, this discussion focuses on the capture element only, and assumes that CO_2_ can be readily disposed of into suitable geological formations close to the facility. Offshore disposal into a depleted natural gas field would also reduce concerns over external costs associated with possible long term leakage of CO_2_ from the reservoir.

#### 3.3.1. Technical Overview of Options

In this analysis, seven CCS cases were examined and compared in terms of effectiveness and cost, and put into a larger context by including four other ways of reducing GHG emissions from the facility. CCS options involve various combinations of pre-*versus* post-combustion capture, central power station and direct drive, and retrofit *versus* new build installation. Table 5 lists each case along with a median estimate of the total capital and operational costs for the facility as equipped. For retrofit options, these figures include lost revenue from down-time, and assume a retrofit date of 2021. As shown, the capital expenditure associated with each case varies considerably. The most expensive of the cases represents a capital cost increase of about 60% of the total installed cost of the business-as-usual reference facility design.

**Figure 1.**
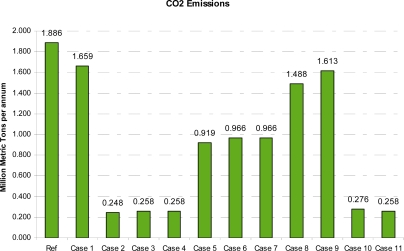
Annual CO_2_ emissions of each case.

Figure 1 shows the annual CO_2_ emissions of each case. Note that all of the CCS cases significantly reduce emissions and improve GHG emissions intensity performance. The reference plant is estimated to produce about 0.25 tCO_2_/t LNG, whereas the CCS designs achieve in the range of 0.03–0.13 tCO_2_/t LNG. Syngas production and pre-combustion capture (Cases 5, 6 and 7) by steam methane reforming have the highest fuel gas use, highest CAPEX and a much poorer emission performance than the post-combustion capture cases (Cases 2, 3 and 4).

#### 3.3.2. Life Cycle Environmental and Economic Sustainability Assessment (EESA)

Using the EESA methodology discussed above, the full life cycle environmental, social and economic sustainability of various CCS and non-CCS cases was evaluated, by considering all of the usual financial parameters (CAPEX, OPEX, energy costs), but also by including the value of various emissions to the atmosphere, notably CO_2_, NOx and SOx. This type of analysis explicitly recognises the key issues associated with a project, and using the common metric of money, allows trade-offs between parameters to be examined across a wide range of possible future conditions. Cases which result in a net overall positive benefit to society as a whole (including the operator), within the limits of the study (in this case LNG production only, not including other parts of the LNG life-cycle) are deemed economic and sustainable. If a case produces less overall benefit to all stakeholders, over its life-cycle, than it costs, when all relevant environmental, social and financial aspects are considered, then it is uneconomic, and unsustainable (even if it is financially profitable to the operator).

Specific assumptions used in the analysis are listed in Table 6. For sensitivity analysis, high and low ranges are also provided for each key parameter. A base case value of USD 25/t CO_2_e was selected For CO_2_, based on the long term average European Trading System price. The high estimate is based on the Stern Review, which set the social cost of carbon at approximately USD 85/t CO2e. Nitrogen oxides (NOx) and sulphur oxides (SOx) emissions result in dis-benefits such as respiratory illness and acid rain. Markets for NOx and SOx emissions limit the volume of NOx and SOx released and allocate the emissions in an economically efficient manner. The largest markets and auctions for these gases are in the USA. Recent EPA spot market prices for SOx and NOx emissions are USD 520/t and USD 640/t, respectively [18]. Clean Air Conservancy are offering offsets for SOx and NOx at approximately USD 1653/t and USD 2646/t respectively [19]. Given the range of recognised values above, median values for SOx and NOx were chosen as USD 521/t and USD 637/t respectively. The analysis was conducted over a 40-year planning horizon, matching the estimated useful life of the facility. Retrofit was assumed to occur in 2021 in all cases. All values are in real 2010 dollars; inflation is not included.

#### 3.3.3. Base Condition Results

**Figure 2.**
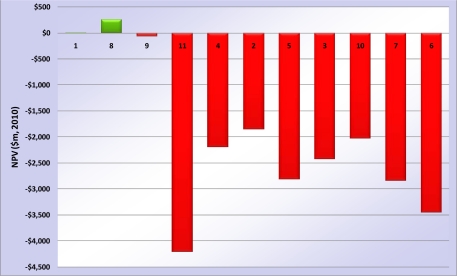
Base condition results—economic NPV compared to reference case (2010 USD m).

The results of the base assessment are presented in Figure 2, with net present values (NPVs) for each option compared to the reference case. Where an option shows positive NPV, it performs that much better than the reference case, over the 40-year life cycle. The results cover the full economic analysis (inclusive of financial and social costs and benefits, and with transfer payments removed). This is a marginal assessment – the revenues generated by the sale of LNG are not included; only the differences associated with GHG management are considered. Options are listed in order of increasing CAPEX from left to right.

Under base conditions, only two of the six options were more economic than the reference case—Case 8 (best-in-class energy efficiency option; NPV + USD 268 million) and Case 1 (CCS of CO_2_ from the reservoir gas stream: NPV +USD 5 million). Case 1 achieves carbon reduction benefits with greater value than the financial costs of achieving them. Case 8 also achieves significant carbon reduction benefits, though the majority of the benefits for this case are from fuel cost savings from more efficient plant design; the combination of these benefits far exceeds the financial costs of achieving them.

It should be stressed that the costs included for the CCS Cases 1–7 are for capture only—and do not include any of the sometimes significant costs for CO_2_ transport and disposal. Equally, none of the potential external costs of sequestration are included (all of which would make CCS *less attractive*, from a full life-cycle environmental, social and economic perspective). Under base conditions, CCS is not economic, even using a low social discount rate and a reasonable median estimate for cost of carbon. In this case, the costs of implementation are simply too high to provide value for society—in fact, all CCS cases, retro-fit and greenfield, are more than a billion dollars worse, in present value terms, than business-as-usual under base case conditions. This is *before* the financial and social costs of sequestration are included. The inference is that society should find other ways of reducing GHG emissions before CCS is deployed on LNG facilities, other than for reservoir CO_2_ stripped from the raw natural gas.

#### 3.3.4. Sensitivity Analysis

Any analysis of this type is inherently subject to uncertainty. Capital and operating costs are planning level estimates suitable for comparison purposes but subject to change. The valuation and estimation of external benefits and certain costs are subject to even larger variations, especially over a 40-year horizon. However, the key to the EESA is to reveal not absolutes in terms of dollars, but better and worse decisions overall, compared to the range of possible decisions that could be made.

From this perspective, sensitivity analysis is important because it allows the overall conclusions of the analysis to be tested across a wide range of parameter inputs. If a decision is favourable or economic over a wide range of parameter inputs, compared to other possible decisions, then despite the overall uncertainty in the actual dollar figures, the decision can be identified as superior among its competitors. This is particularly useful when examining the sustainability of options. By definition, sustainability is concerned with the future, which is inherently uncertain. By varying key input parameters over a wide but reasonable range, the implications of a range of possible futures can be examined. The ranges of values for key parameters for this assessment are presented in Table 6.

A fully interactive real time analysis tool, EcoNomics™ DELTΔ2™ [20], has been used to examine the effect of key parameters, across their full range, for each option. The NPV results are calculated for each value of each parameter, discretised across its full range, against every other possible combination of the other values, for each case. This in effect provides a database of every possible NPV result for each case, in which each result is considered to be equi-probable.

Table 7 shows the proportion of all conditions where a case is the most economic, or second most economic case, considering all possible combination of all values across their full range. Under approximately 92% of all possible combinations of conditions, Case 8 is the most economic, followed by Case 1 which is the most economic option under approximately 8% of all possible combinations of conditions. Case 1 is the second most economic option under over 73% of all possible combinations of conditions.

Fuel gas savings are the most significant contributor to the positive economics of Case 8, far exceeding the financial costs of achieving them. Associated benefits from GHG reductions are also significant. Increasing energy costs and a higher cost of carbon further increase the positive NPV achieved by this case. Case 1 is also economic under a wide range of possible future conditions, mostly driven by the GHG reductions achieved by this option compared to the reference case. It is the cheapest way to reduce GHG emissions. NOx and SOx values did not contribute significantly to the costs and/or benefits of each option.

#### 3.3.5. Implications

For this facility, the analysis reveals that the environmentally, socially and economically optimum approach would be to implement Case 8 (best-in-class energy efficiency design across the facility) and Case 1 (CCS of CO_2_ contained in the reservoir gas), either alone or in combination. The combined base condition economic NPV of these cases is USD 272 million better than business as usual. With rising energy costs and the increasing likelihood of rising carbon costs, each of the cases assessed delivers increasing overall economic benefits to society. This is an important finding with respect to longer-term capital investment decisions. Further insight can be had from examining narrower ranges of future possible conditions. In a “socially-minded” world, where carbon is assumed to be priced at greater than USD 50/tCO_2_e, and where the maximum discount rate considered is 6%, Case 8 is best under 95% of conditions. In a more “commercially-minded world”, where carbon price is assumed to be always lower than USD 50/tCO_2_e, and discount rate is higher than 6%, Case 8 is still the most economic and sustainable under 79% of the remaining range of conditions over 40 years.

#### 3.3.6. Option Value of Being Carbon Capture Ready

**Figure 3.**
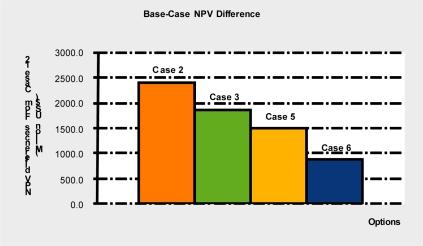
Base NPV differences between capture ready retrofit and capture unready plants.

Making LNG plants carbon capture ready enables retrofitting at a lower cost at some point in the future, at the expense of a relatively small upfront investment [21–23]. The main requirements for being carbon capture ready are that sufficient space and access are provided for future deployment of capture equipment and systems. Figure 3 shows the difference in NPV over 40 years, at base values, between the CCS retrofit options on a capture ready plant and on a plant that is not capture ready (a plant where no provision for retrofit has been made), of similar overall design. Clearly, retrofitting a capture ready plant with CCS technology is more economic than on a carbon capture unready plant, no matter what year the retrofit is made. As shown above, Case 2 is the best of the CCS retrofit options.

**Figure 4.**
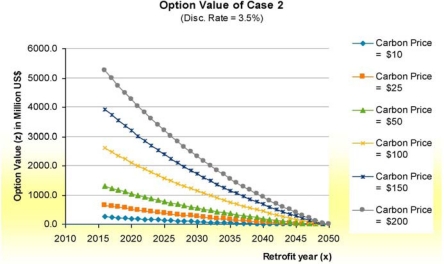
Option value of being capture ready for Case 2, base conditions.

The option value of being capture ready was also examined (the value of the capture ready pre-investment in implementing retrofit in a given year). Figure 4 shows the option value for being capture ready in implementing Case 2 (the best of the retrofit CCS options). As shown, the capture ready case value is highly dependent on carbon price and the timing of the retrofit. The longer one waits to undertake the retrofit, the less the option is worth, as one approaches the end of life of the facility (retrofitting in 2049 leaves only one year of carbon cost savings to recoup the investment).

#### 3.3.7. Carbon Price Trigger Points for CCS

The analysis provides an indication of the threshold carbon price at which the decision to implement various carbon reduction measures would occur. Under base conditions, assuming social (3.5%) and commercial (10%) discount rates, the carbon price thresholds (in 2010 USD) that would trigger action are shown in Table 8. Note than in each sense, the first two cases to be deployed are Cases 8 and 1. Case 8 should be done now—it is already economically positive. The best of the CCS cases (Cases 4 and 2) are not economically or socially viable unless carbon prices/values are higher than USD 100/tCO_2_e. Also recall that the financial and external costs of transport and storage would have to be added to the figures shown, which would in all cases raise the threshold value for CCS implementation. In the context of current estimates of the social cost of carbon, CCS does not appear to be viable for managing emissions from facility liquefaction and compression.

## 4. Discussion

The examples presented above examine the wider environmental and economic implications of applying CCS in various applications. CCS is an expensive proposition, no matter how it is applied, both in terms of up-front capital costs, and in terms of the significant amounts of energy required to capture CO_2_. Because of this, it is important that CCS performance be compared to other ways of reducing GHG emissions within a plant or portfolio of assets, if they exist. Strategically, this serves to put CCS into overall context as just one of many possible ways that the GHG intensity of an operation can be reduced.

In the case of existing coal-fired power stations, CCS may be one of the only options available for substantially reducing GHG emissions. In this case the question becomes which of a wide variety of CCS options available provides the best long term environmental, social and financial outcome. As the world moves to take action on climate change, and carbon prices rise in future, CCS will become increasingly attractive from a financial perspective. In other industries, such as LNG, a number of other alternatives are available for carbon emission reduction. Designing for energy efficiency and including maximum use of waste heat, may provide distinctly economic and sustainable alternatives. Under a wide range of future likely conditions, sequestering CO_2_ from reservoir gas makes good economic sense and is a sustainable proposition today.

The analysis presented herein suggests that while all CCS options are not created equal (some are far more economic and sustainable than others), overall, CCS (other than capturing CO_2_ directly from the raw reservoir gas) is unlikely to be economic or sustainable at carbon prices much below about USD 75/t CO_2_e, which is close to current estimates of the social cost of carbon. At Stern’s USD 85/t CO_2_e estimate, CCS is an economic choice right now for certain types of applications, as far as society is concerned. However, operators will have little incentive to deploy CCS unless the social value of carbon is wholly or partially reflected in a regulated price on carbon.

From a financial perspective, CCS costs need to come down significantly to make it viable in the near future for most applications, even as carbon prices are put in place around the world. Increased funding for research and development of new breakthroughs in carbon capture technology could substantially improve the viability of CCS in the coming years. Carbon sequestration also carries with it significant long-term cost and legislative and environmental risks which would also have to be factored into a complete analysis.

In some jurisdictions, there is the possibility that CCS will be required, by law, policy or regulation. If, for instance, a company self-regulates and sets a policy of zero carbon emissions, CCS may become one of the only ways to achieve this. It is also possible that carbon prices may rise to the kinds of levels which are needed for CCS to be justified from an overall environmental, social and economic cost-benefit point of view. The social cost of carbon continues to increase with each year of global inaction, as the concentrations of GHGs in the atmosphere steadily rise. As the social cost of carbon rises, CCS becomes an increasingly more economic proposition.

If we must retrofit, retrofitting a capture ready plant is a significantly more economic proposition than retrofitting a carbon capture ignorant plant. The cost savings of being carbon capture ready (with a relatively minimal pre-investment cost), compared to having to retrofit a plant that is not capture ready, are significant.

## 5. Conclusions

While there are many different ways for the power and energy industries to eliminate carbon emissions to atmosphere, they come at radically different costs, and produce different benefits to society. Worldwide, CCS is being intensely examined as a way of reducing emissions from fossil-fuel burning or producing operations. While full-scale deployment of CCS globally is still in its infancy, several successful long-term projects are underway (notably in the natural gas sector), and a suite of demonstrations projects have begun in various parts of the world, in various industry sectors. A wide variety of capture technologies are available, with widely differing capital costs, operational costs, energy requirements, and performance. Safe long-term geological sequestration requires detailed understanding of reservoir characteristics and appropriate long term institutional controls and monitoring. The external social and environmental costs of large scale releases from storage sites could be significant, if these eventuate, but thorough risk management in terms of site selection, monitoring and reservoir design have proven capable of significantly if not completely reducing the likelihood of such releases. The environmental and economic sustainability of CCS is a function of many factors, including the timing of deployment, the value placed on carbon, fuel and energy prices, the costs of disruptions to the business during retrofit, and the external costs of other associated air emissions and possible releases from storage sites. Analysis of three examples shows that all CCS is not created equal, and that industries should examine CCS in the context of other alternatives that exist to reduce emissions within their business, either at individual facilities or within a larger portfolio of assets. In addition, the wider external costs and benefits must be taken into consideration if the real value, and therefore the environmental and economic sustainability, of the action is to be understood.

## Figures and Tables

**Table 1.** Costs and benefits of acting to mitigate climate change.

**Study**	**Costs of Action**	**Benefits of Action**	**Comment**
Stern (2006) [6]	+1 to −1% Global Product to 2030; approximately USD 250 bn/year over this period	Prevention of the loss of 5 to 20% of Global Product now and forever	Costs to the global economy of inaction are permanent, irreversible, and catastrophic
Garnaut (2008) [7]	−0.8% initial change in annual Australian Gross National Product (GNP), followed by−0.2 to −0.0% change in annual Australian GNP to 2050	0.0 to 0.2% increase in annual Australian GNP in second half of this century.	From 2050, mitigation adds to the growth rate of the Australian economy, as, at the margin, more new climate change damages are avoided than new mitigation costs added. By the end of the century, Australian GNP is higher than it would have been without mitigation.
IEA and OECD (2009) [8]	USD 4.1 tn over the next 20 years	Savings in fuel costs alone of over USD 7 tn; stabilisation of GHG concentrations in the atmosphere above 550 ppm CO_2_e	Significant expenditure on R&D expected to bring overall cost of stabilisation down; CCS to play key role in overall mitigation globally

**Table 2.** Capture technologies considered.

**Technology**	**Technology Type**	**Application to PCC**	**Commercial Status**
Monoethanolamine	Chemical solvent	Yes	Commercial
Chilled Ammonia	Chemical solvent	Yes	Pilot
KS Solvents	Chemical solvent	Yes	Pilot
Aqueous Ammonia	Chemical solvent	Yes	Pilot
Methyl Diethanolamine	Chemical solvent	No	Commercial
Diethanolamine	Chemical solvent	No	Commercial
Selexol	Physical solvent	No	Commercial
Sulfinol	Mixed chemical-physical solvent	No	Commercial

**Table 3.** Capture performance.

**Option**	**Emissions to Atmosphere (Mtpa CO_2_e)**	**Net Power Output at Boiler MCR (MW)**	**Emissions Captured (Mtpa CO_2_e)**
Base case (Current operations)	2.0	425	0
Option 1 (5,500 tpd)	0.8	325	2.0
Option 2 (8,200 tpd)	0.25	275	2.7

**Table 4.** Financial cost summary, CCS retrofit (millions 2008 USD/tCO_2_e).

	
	**Option 1**	**Option 2**
Least-preferred scenario	120	145
Preferred scenario	71	90

**Table 5.** GHG management cases and cost data (million US $ 2010).

**Case**	**Description**	**CAPEX**	**Annual OPEX**
Ref.	Standard LNG facility currently in operation, business-as-usual, no CCS fitted	4,400	160
1	Carbon capture from the reservoir gas only + CCS	4,500	160
2	Retrofit post-combustion capture (liquefaction), aero-derivative gas turbine drive + CCS	5,700	160
3	Retrofit natural gas combined cycle (GTCC) central power generation, post-combustion capture, electric motor compressor drives in liquefaction trains + CCS	6,000	250
4	Option 3, but greenfield	5,500	160
5	Retrofit local pre-combustion capture, aero-derivative gas turbine drive + CCS	6,000	160
6	Retrofit central GTCC, pre-combustion capture, electric motor drives in liquefaction trains + CCS	6,500	230
7	Option 6, but greenfield	6,100	160
8	Local combined cycle power, best-in-class energy efficiency, additional gas turbine waste heat recovery + part steam turbine direct compression drives	4,600	170
9	Central combined cycle power, best-in-class, electric motor compressor drives in liquefaction trains	4,800	160
10	Surplus power generation, exporting to grid, and	6,000	165
11	Buy power from de-carbonised grid.	5,200	160

**Table 6.** Assumptions for key parameters.

**Parameter**	**Low Value**	**Base Value**	**High Value**
Discount rate	3.0%	3.5%	10%
Fuel gas price	USD 1/mmbtu	USD 3/mmbtu	USD 5/mmbtu
LNG price	-	USD 7.50/mmbtu	-
Power on-sale price (Opt 10)	USD 40/MWh	USD 50/MWh	USD 100/MWh
Power purchase price (Opt 11)	USD 50/MWh	USD 62.50/MWh	USD 125/MWh
Carbon cost	USD 0 /tCO_2_e	USD 25/tCO_2_e	USD 85/tCO_2_e
SOx price	USD 0/t	USD 521/t	USD 1,860/t
NOx price	USD 0/t	USD 637/t	USD 2,360/t

**Table 7.** Proportion of conditions where a case is the most economic.

**Option**	**Best case**	**2nd Best case**
Option 8	91.6%	8.4%
Option 1	7.5%	73.0%
Option 10	1.0%	2.9%
Option 9	0.0%	15.7%

**Table 8.** Carbon price threshold for capture.

**Case**	**Social (*i* = 3.5%)**	**Commercial (*i* = 10%)**
8 – Energy efficient design	USD 0	USD 0
1 – CCS reservoir CO_2_	USD 20	USD 25
9 – Centralised power, efficient design	USD 38	USD 120
10 – Surplus power generation	USD 85	USD 100
2 – Post-combustion CC retrofit	USD 100	USD 145
4 – Greenfield NGCC post-combustion retrofit	USD 116	USD 150
